# Long‐Term Recurrence‐Free Outcome After Doxycycline Pleurodesis With Pulmonary Rehabilitation and Nutritional Support in an Underweight Patient With COPD: A Case Report

**DOI:** 10.1002/rcr2.70675

**Published:** 2026-08-28

**Authors:** Kemas Rakhmat Notariza, Prasenohadi Pradono, Siti Chandra Widjanantie, Elfina Rachmi, Agus Dwi Susanto

**Affiliations:** ^1^ Department of Pulmonology and Respiratory Medicine Faculty of Medicine Universitas Indonesia—Persahabatan Hospital National Respiratory Center Jakarta Indonesia; ^2^ Department of Physical Medicine and Rehabilitation Faculty of Medicine Universitas Indonesia—Persahabatan Hospital National Respiratory Center Jakarta Indonesia; ^3^ Department of Clinical Nutrition Faculty of Medicine Universitas Indonesia—Persahabatan Hospital National Respiratory Center Jakarta Indonesia

**Keywords:** chronic obstructive pulmonary disease, nutrition, pleurodesis, pneumothorax, rehabilitation

## Abstract

Secondary spontaneous pneumothorax (SSP), a complication of chronic obstructive pulmonary disease (COPD), recurs more frequently in underweight patients. This case report describes a 63‐year‐old malnourished man with COPD presenting with recurrent left‐sided SSP shortly after hospital discharge for a previous episode. Following chest tube reinsertion and complete lung re‐expansion, chemical pleurodesis was performed using 1000 mg doxycycline in saline. Transient pleuritic pain was controlled with intravenous ketorolac and intrapleural lidocaine. No other immediate or delayed adverse events occurred. In parallel, pulmonary rehabilitation, including breathing exercises, posture correction, moderate‐intensity endurance and inspiratory muscle training, was initiated alongside nutritional repletion with a respiratory‐specific oral nutritional supplement. Over a 22‐month follow‐up, the patient remained free of SSP recurrence, with improved functional capacity and weight gain. This report describes a long‐term recurrence‐free outcome after doxycycline pleurodesis combined with pulmonary rehabilitation and nutritional support in an underweight patient with COPD.

## Graphical abstract

Recurrent secondary spontaneous pneumothorax (SSP) in chronic obstructive pulmonary disease (COPD) is associated with structural lung damage and underweight status. In this case, doxycycline pleurodesis combined with pulmonary rehabilitation and nutritional support was associated with sustained lung re‐expansion and a 22‐month recurrence‐free outcome.

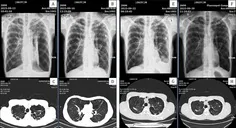

## 1 Introduction

Chronic obstructive pulmonary disease (COPD) is characterised by progressive airflow limitation [1]. Secondary spontaneous pneumothorax (SSP) in COPD is associated with significant morbidity and mortality. The 2023 British Thoracic Society (BTS) guideline recommends chest drainage for acute symptomatic SSP and considers chemical pleurodesis in patients with significant deterioration or high risk of recurrence [2]. In COPD, recurrent SSP is common, and underweight status further escalates recurrence risk [3]. Pulmonary rehabilitation and nutritional support improve functional capacity and muscle mass in COPD [1]. This report describes a long‐term recurrence‐free outcome after doxycycline pleurodesis with pulmonary rehabilitation and nutritional support in an underweight COPD patient with recurrent SSP.

## 2 Case Report

**FIGURE 1:**
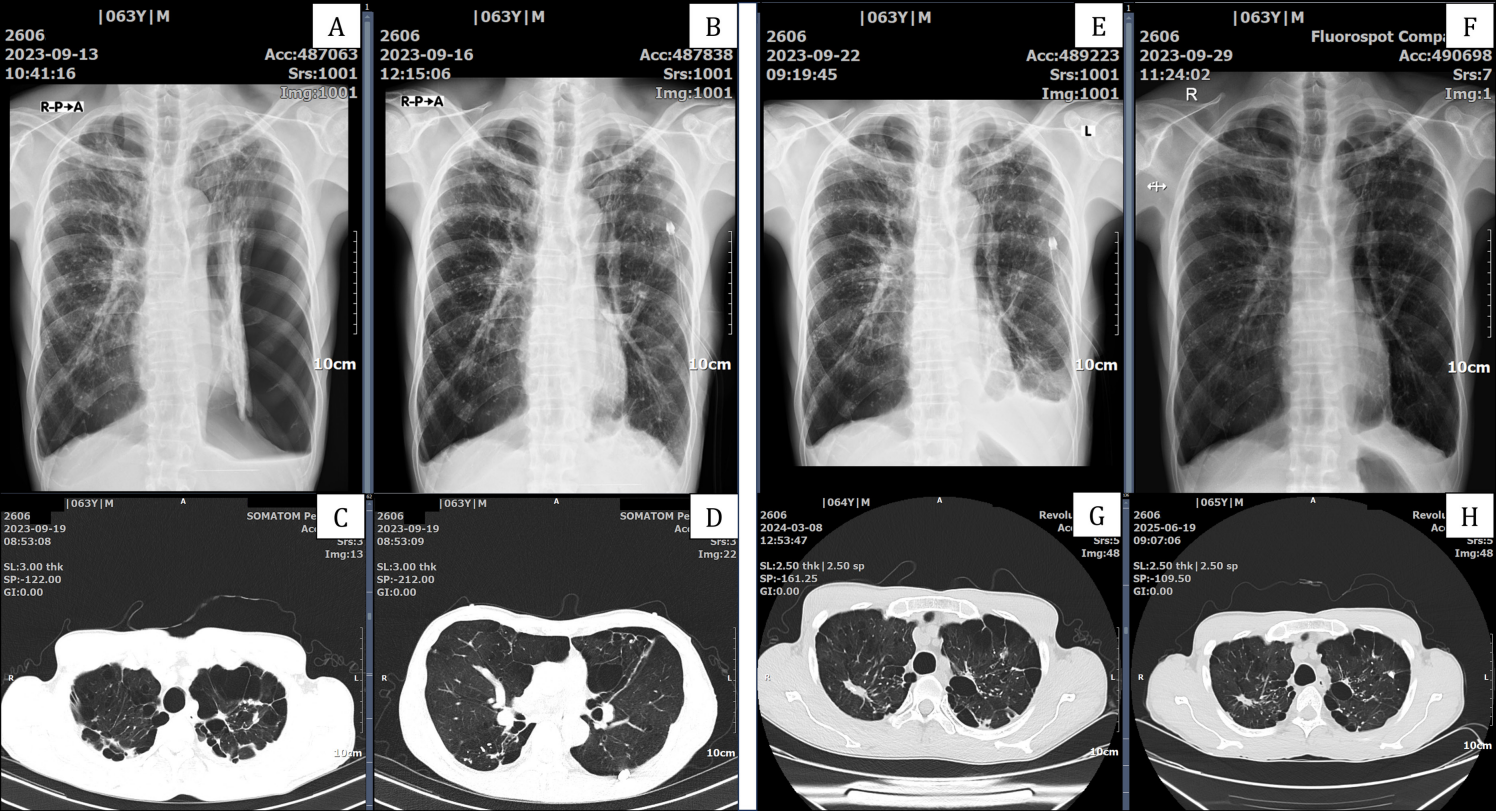
Serial chest imaging studies: (A) chest radiograph on admission; (B) 3 days after ICD insertion; (C–D) chest HRCT showing extensive bilateral bullous emphysema; (E) 2 days after pleurodesis; (F) 1 week post‐pleurodesis; (G) chest HRCT 6 months after pleurodesis; and (H) chest HRCT 20 months post‐pleurodesis.

A 63‐year‐old man with Group E COPD (baseline post‐bronchodilator FEV_1_ 53% predicted, mMRC 1, 36 pack‐years smoking history) presented with worsening dyspnea for 3 days. Group E reflected hospitalisation for severe COPD exacerbation 9 months earlier, when his first right‐sided SSP occurred. He used tiotropium bromide 2.5 mcg (2 puffs once daily) without long‐term oxygen therapy or other comorbidities. He was discharged 5 days before the present admission following management for his first left‐sided SSP. His body mass index (BMI) was 16.9 kg/m^2^. Chest radiograph confirmed a recurrent left SSP (Figure 1A).

A 24‐Fr intercostal drain (ICD) was inserted and connected to a water‐sealed drainage (WSD) system without suction. Complete lung re‐expansion was achieved within 3 days without persistent air leak (Figure 1B). High‐resolution computed tomography (HRCT) revealed extensive bilateral bullous emphysema (Figure 1C,D). Given the rapid SSP recurrence and structural vulnerability, bedside doxycycline pleurodesis (1000 mg in 50 mL saline) was performed after obtaining informed consent, administering intravenous ketorolac and intrapleural lidocaine. Following repeated body rotations, the ICD was clamped for 48 h and removed after minimal drainage without air leakage (Figure 1E). Transient pleuritic pain was controlled; no other immediate adverse events were observed during hospitalisation. Table 1 summarises the timeline.

**TABLE 1:** History of clinical events.

Timeline	Clinical events
9 months prior	Hospitalisation for COPD exacerbation, during which the first episode of right‐sided SSP developed and resolved with ICD insertion.
10 days prior	First episode of left‐sided SSP; insertion of ICD, connected to a WSD system without suction.
5 days prior	Discharged after complete lung re‐expansion on the first episode of left‐sided SSP.
Day 0 (admission)	Presentation with worsening dyspnea (3 days); diagnosed with recurrent left SSP. Left ICD inserted, connected to a WSD system without suction.
Day 3	Complete lung re‐expansion confirmed via chest radiograph. Chest HRCT showed extensive bullous emphysema.
Day 4	Bedside chemical pleurodesis using 1000 mg doxycycline. Initiation of in‐hospital pulmonary rehabilitation and nutritional therapy.
Day 6	Left ICD removed 48 h after pleurodesis. Patient discharged with maintenance inhalers, as well as outpatient pulmonary rehabilitation and nutrition programme.

Abbreviations: HRCT, high‐resolution computed tomography; ICD, intercostal drain; SSP, secondary spontaneous pneumothorax; WSD, water‐sealed drainage.

During hospitalisation, nutritional therapy targeted total daily energy expenditure (TDEE) 1902 kcal/day and 1.5 g/kg/day protein (75 g), using regular meals (1500 kcal/day) and two daily servings of a respiratory‐specific oral nutritional supplement (ONS; 240 kcal, 12.5 g protein, 1:1 carbohydrate‐to‐fat ratio and 400 mg omega‐3 per serving). He achieved over 80% of his caloric target. Concurrently, supervised pulmonary rehabilitation comprised inpatient sessions (3–5 times/week, 30–60 min/session) including: active cycle of breathing technique (ACBT) with huffing; diaphragmatic and pursed‐lip breathing (PLB); postural correction; upper‐extremity stretching; and below‐shoulder strengthening exercise. Upon discharge, indacaterol 150 mcg (1 puff once daily) was added.

Aerobic training progressed from 5 to 10 min of treadmill walking at 40%–60% heart rate reserve to 30–60 min of daily home walking. Progressive inspiratory muscle training (IMT) used a threshold device, with vital signs and Borg dyspnea scale monitored for safety. Longitudinal functional measurements were obtained during stable outpatient follow‐up. The 6‐min walk test (6MWT) used standardised European Respiratory Society (ERS)/American Thoracic Society (ATS) instructions and a 15‐m corridor based on the Indonesian anthropometric reference protocol [4, 5]. Percentage‐predicted 6MWT distance, estimated maximum oxygen consumption (VO_2_max) and metabolic equivalent of tasks (METs) were calculated using equations detailed in Table 2 [5, 6]. Peak cough flow (PCF) and peak flow rate (PFR) were measured upright with a handheld peak‐flow metre during outpatient rehabilitation, using forceful cough and forced expiratory manoeuvres, respectively. The highest value of three acceptable attempts was recorded.

**TABLE 2:** Longitudinal clinical, respiratory and functional outcomes during 22‐month follow‐up.

Follow‐up period	Body weight (kg)	BMI (kg/m^2^)	Post‐BD% predicted FEV1 (%)	6MWT distance (m)	% Predicted 6MWT distance (%)	VO_2_max (mL/kg/min)	METs	PCF (L/min)	PFR (L/min)	Dyspnea measure (mMRC)
September 2023 (Baseline)	50	16.9	53.0	395	67.8	15.16	4.33	260	240	1
November 2023 (Month 2)	51.5	17.4	—	500	85.7	17.22	4.92	210	260	1
February 2024 (Month 5)	53	17.9	—	495	84.7	17.12	4.89	250	295	0
March 2024 (Month 6)	53	17.9	57.0	454	77.7	16.30	4.66	300	280	0
June 2024 (Month 9)	54.5	18.3	—	495	84.7	17.12	4.89	295	285	1
October 2024 (Month 13)	56	18.9	64.0	510	87.3	17.40	4.97	300	300	1
January 2025 (Month 16)	57	19.3	—	500	85.0	17.20	4.91	240	250	1
April 2025 (Month 19)	58	19.6	—	494	84.0	17.10	4.89	290	285	0
July 2025 (Month 22)	61	20.6	62.0	510	87.3	17.40	4.97	265	265	0

*Note:* Percentage‐predicted 6MWD was calculated using the Indonesian anthropometric equation predicted 6MWD = 586.254 + 0.622 × body weight (kg) − 0.265 × body height (cm) − 63.343 × sex +0.117 × age (years), with sex coded as 0 for male and 1 for female [5]. The VO_2_max was estimated using the Cahalin equation: VO_2_max (mL/kg/min) = 0.006 × 6MWD (feet) + 7.38. The METs were calculated as VO_2_max/3.5 [6].

Abbreviations: 6MWT, 6‐min walk test; BD, bronchodilator; BMI, body mass index; FEV1, forced expiratory volume in 1 s; METs, metabolic equivalent of tasks; mMRC, modified Medical Research Council; PCF, peak cough flow; PFR, peak flow rate; VO_2_max, estimated maximum oxygen consumption.

At 1‐week follow‐up, lung expansion was maintained without effusion (Figure 1F). Over 22 months, no SSP recurred. Longitudinal parameters showed sustained weight gain, improved exercise capacity and health status (Table 2). No delayed pleurodesis‐related adverse events were identified. Follow‐up chest HRCT confirmed stable emphysema without SSP recurrence (Figure 1G,H).

## 3 Discussion

For symptomatic SSP, the 2023 BTS guideline recommends ICD insertion. Although elective surgery (video‐assisted thoracoscopy surgery [VATS] or thoracotomy) should be considered in the second ipsilateral or first contralateral pneumothorax, this patient was deemed unfit for surgery due to extensive bilateral bullous emphysema and limited respiratory reserve (baseline FEV_1_ 53% predicted) [2].

In severe COPD with SSP‐related decompensation, chemical pleurodesis should be considered to prevent recurrence, even during or after the first episode [2]. Doxycycline promotes pleural symphysis through inflammation and pleural apposition. Compared to talc slurry, doxycycline provides comparable efficacy, lower complication risk, cost‐effectiveness and bedside accessibility through ICD [3].

While pleurodesis addresses the structural pleural defect, systemic and functional optimisation remains relevant in underweight COPD patients. Low body weight is a predictor of pleurodesis failure [3]. Nutritional support yielded sustained weight gain and improved functional outcomes. Adequate protein intake may support recovery by providing metabolic substrates, while the respiratory‐specific ONS also contained omega‐3 [1]. Although pleural‐healing causality cannot be proven from one case, nutritional repletion was an important component of clinical optimisation in this underweight patient.

Structured pulmonary rehabilitation was introduced after clinical stabilisation and continued during follow‐up. The programme aimed to improve breathing control, airway clearance, dyspnea, exercise tolerance and functional status. Controlled breathing techniques (PLB and diaphragmatic breathing) may reduce dynamic hyperinflation in COPD [1], while low‐pressure huffing, below‐shoulder upper‐extremity movements, vital sign monitoring and Borg dyspnea assessment were used to maintain safety after pleurodesis. Together with doxycycline pleurodesis, this multidisciplinary approach was associated with a favourable 22‐month recurrence‐free outcome and functional improvement.

In conclusion, doxycycline pleurodesis could be a safe, accessible intervention for obliterating the pleural space in COPD patients with recurrent SSP and prohibitive surgical risks. In this underweight patient, doxycycline pleurodesis combined with pulmonary rehabilitation and nutritional support was linked to a sustained recurrence‐free outcome, weight gain and improved functional status.

## Author Contributions

All authors collaborated in the conception of this case report and data interpretation for the work. K.R.N. was responsible for data acquisition and work drafting. K.R.N., P.P., S.C.W. and E.R. were directly involved in the patient care. P.P., S.C.W., E.R. and A.D.S. critically reviewed the manuscript. All authors approved the final version of the manuscript.

## Funding

The publication fee for this article was supported by the CRU, Persahabatan Hospital National Respiratory Center, Jakarta, Indonesia. The funder had no role in data collection, management, analysis or interpretation of the data; nor in the preparation, review or approval of the manuscript.

## Consent

The authors declare that written informed consent was obtained for the publication of this manuscript and accompanying images using the consent form provided by the Journal.

## Conflicts of Interest

The authors declare no conflicts of interest.

## Data Availability

The data that support the findings of this study are available on request from the corresponding author. The data are not publicly available due to privacy or ethical restrictions.
